# A systematic review and meta-analysis on computed tomography angiography mapping for deep inferior epigastric perforator flap breast reconstruction

**DOI:** 10.3389/fonc.2025.1600476

**Published:** 2025-09-17

**Authors:** Roshan S. Rupra, Francesca Ruccia, Kian Daneshi, Fatema Aftab, Yousif F. Yousif, Gul R. Khan, Sina Dehnadi, Yaqoob H. AlSaidi, Nicola Maggialetti, Giovanni Lorusso, Maria Yan, Ankur Khajuria

**Affiliations:** ^1^ Department of Plastic Surgery, Royal Devon University Healthcare NHS Foundation Trust, Exeter, United Kingdom; ^2^ Department of Plastic Surgery, The Royal Marsden NHS Foundation Trust, London, United Kingdom; ^3^ School of Medicine and Population Health, The University of Sheffield, Sheffield, United Kingdom; ^4^ Department of Bioengineering, Imperial College London, London, United Kingdom; ^5^ Department of Radiology, Cambridge University Hospitals, Cambridge, United Kingdom; ^6^ Department of Emergency Medicine, West Hertfordshire Teaching Hospitals NHS Trust, Watford, United Kingdom; ^7^ Department of Surgery, James Paget University Hospital NHS Foundation Trust, Great Yarmouth, United Kingdom; ^8^ Department of Neurosurgery, Suhar Hospital, Ministry of Health Oman, Muscat, Oman; ^9^ Interdisciplinary Department of Medicine, Section of Radiology and Radiation Oncology, University of Bari “Aldo Moro”, Bari, Italy; ^10^ Department of Surgery, University of Connecticut, Farmington, CT, United States; ^11^ Department of Surgery & Cancer, Imperial College London, London, United Kingdom

**Keywords:** DIEP, CTA, operative time, complication rate, flap loss

## Abstract

**Background:**

Breast cancer remains the most prevalent cancer among women globally, necessitating effective reconstructive options post-mastectomy. The deep inferior epigastric perforator (DIEP) flap is the gold standard for autologous breast reconstruction, though anatomical variability of perforators presents surgical challenges. Computed tomography angiography (CTA) has been proposed to enhance preoperative planning and reduce operative time. The aim of this study is to identify how CTA affects surgical outcomes in autologous breast reconstruction.

**Methods:**

A systematic review and meta-analysis (PROSPERO: CRD42024596646) were conducted per PRISMA guidelines. A comprehensive search of six databases identified studies comparing CTA with non-CTA imaging for DIEP flap reconstruction. Primary outcomes included operative time and flap loss rates. Risk of bias was assessed using ROBINS-I and RoB2, with quality appraised via AMSTAR-2 and GRADE.

**Results:**

Eighteen studies (3870 patients, 4283 flaps) were included. CTA guidance reduced unilateral flap operative time (mean 304.98 min vs. 390.18 min, CI −12.9 to 5.7; P = 0.2367), as well as partial and total flap loss rates (OR: 0.26, 95% CI: 0.14–0.47; OR: 0.30, 95% CI: 0.13–0.68). High heterogeneity (I² = 98.7%) limited generalizability. Prior reviews showed limitations in study design integrity, whereas this study achieved a high-confidence rating.

**Conclusions:**

Preoperative CTA improves surgical outcomes in DIEP flap reconstruction, though evidence quality is variable. Future research should compare CTA with MRA, assess cost-effectiveness, integrate AI-assisted imaging, and explore MRI-based protocols for optimized preoperative planning in microsurgical breast cancer reconstruction and enhanced oncologic care delivery.

**Systematic review registration:**

https://www.crd.york.ac.uk/PROSPERO/view/CRD42024596646, idenitifier CRD42024596646.

## Introduction

Among autologous breast reconstruction techniques, the deep inferior epigastric perforator (DIEP) free flap is considered the gold standard due to its high success rates, low complication rates, shorter hospital stays and superior long-term cosmetic and quality of life outcomes compared to other techniques (1–4). By optimizing patient selection and surgical planning, preoperative imaging such as CTA may contribute to more efficient cancer care delivery, reduced complication rates, timely initiation of adjuvant therapies, and improved overall outcomes in breast cancer treatment pathways.

A critical determinant of DIEP flap efficiency and safety is the precise identification of suitable perforators. Anatomical variability of perforating vessels from the deep inferior epigastric artery can prolong dissection time and increase the risk of complications during DIEP flap procedures. Preoperative mapping, particularly using computed tomography angiography (CTA), has been shown to improve surgical planning and reduce surgical time, enabling surgeons to select optimal vessels and tailor flap design. Several single-institution series suggest that CTA guidance reduces operative and ischemia times and may lower flap-loss rates (5, 6). Given that prolonged operative time is an independent risk factor for complications, including flap failure, as demonstrated in the ACS-NSQIP study with over 108,000 patients strategies that streamline intraoperative planning have the potential to improve both clinical outcomes and health-care efficiency (7, 8). Indeed, reducing surgical complications is closely linked to shorter inpatient stays and higher patient satisfaction (9).

Despite these promising observations, the evidence base for pre-operative perforator mapping and its quality is equivocal. To address this gap, we conducted a comprehensive, methodologically rigorous systematic review and meta-analysis to evaluate the impact of preoperative CTA-based perforator mapping on operative time and clinical outcomes in DIEP breast reconstruction. We also appraised the quality of the evidence to develop evidence-based recommendations that guide decision-making and optimize patient outcomes.

## Methods

This systematic review and meta‐analysis was conducted in accordance with the Cochrane Handbook for Systematic Reviews of Interventions and reported per the PRISMA 2020 guidelines (10, 11). The protocol was prospectively registered on PROSPERO (CRD42024596646) to ensure transparency and rigor (12). To appraise the quality of existing reviews, we applied the AMSTAR-2 tool in a comparative manner (13).

### Search strategies

Database searches were conducted on 25th September 2024 across PubMed/MEDLINE, EMBASE, the Cochrane Central Register of Controlled Trials (CENTRAL), the Science Citation Index, and Google Scholar. The search strategy, detailed in the Appendix (see **Supplementary File 1**), combined Medical Subject Headings (MeSH) with free-text keywords using Boolean operators. We limited inclusion to peer-reviewed, English-language studies. Additionally, the reference lists of all included studies were screened through citation chaining to identify further relevant publications.

### Study eligibility - inclusion/exclusion criteria

Inclusion criteria:

- All ages.
- All studies with patients that had undergone DIEP Breast Reconstruction and had CT angiographic mapping done pre-operatively.
- All studies with a non-CT angiographic control arm (i.e. no imaging, ultrasound, magnetic resonance, etc.).
- Articles published in peer-reviewed academic journals with available full-text articles.

Exclusion criteria:

- Studies with patients that had only undergone breast reconstruction with techniques other than DIEP flap.
- Studies with no pre-operative CT angiographic mapping.
- Abstracts.
- Case reports.
- Animal studies.

### Identification and selection of studies

Search results were imported into Rayyan (Cambridge, MA, USA), where duplicate records were removed prior to screening. A two‐stage selection process was then carried out independently by five reviewers (R.S.R., G.R.K., Y.A.S., Y.Y., and G.L.) using predefined eligibility criteria.

Stage 1: Titles and abstracts were screened for relevance. Discrepancies between reviewers were resolved through discussion, and any remaining conflicts were adjudicated by an independent author (M.Y.). Studies of uncertain eligibility proceeded to full‐text review.

Stage 2: Full‐text articles deemed potentially eligible were independently assessed by the same reviewers. Persistent disagreements were resolved by consensus, with M.Y. providing the tie‐breaking decision when necessary. We also performed citation chaining of all included articles to capture additional relevant reports.

**Figure 1:**
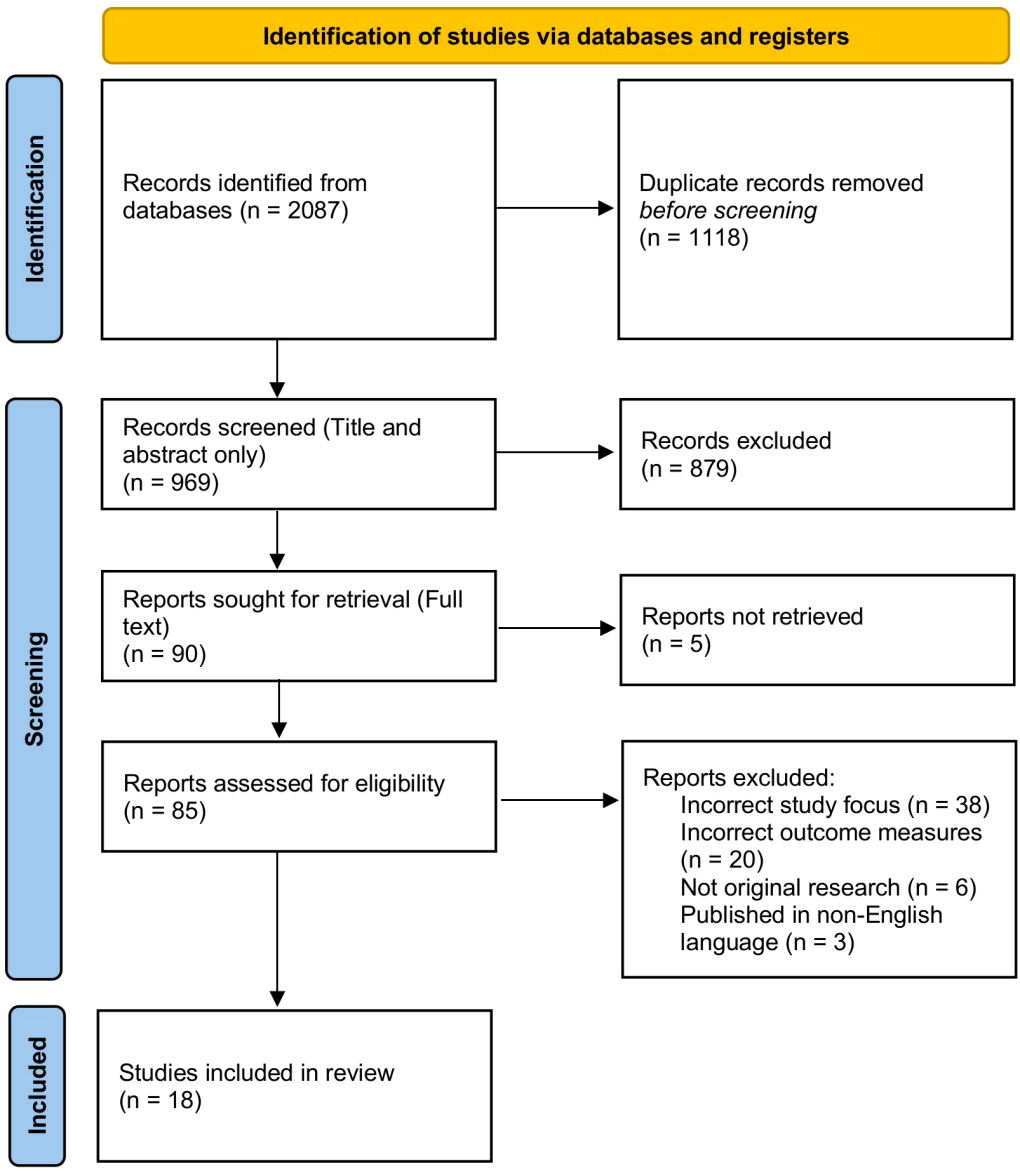
PRISMA flowchart of study inclusion and exclusion.

A flow diagram summarizing the search results and screening outcomes is presented in **Figure 1**.

### Data extraction

Two authors (R.S.R. and F.A.) independently extracted data from all eligible full-text articles using a pre‐piloted, standardized data collection form. Any discrepancies were reconciled by discussion or, if necessary, adjudicated by a third independent author (K.D.). In accordance with AMSTAR-2 guidelines, original study authors were contacted to clarify missing or ambiguous information. Extracted variables encompassed key study characteristics (first author, publication year, country), sample size, operative metrics (total procedure time, flap‐harvest time, ischemia time), and clinical outcomes (partial and total flap‐loss rates).

### Risk of bias and quality assessment

For risk of bias assessment, two authors independently evaluated observational studies using Cochrane’s Risk of Bias in Non-Randomized Studies - of Interventions (ROBINS-I) tool (14). Randomized studies were assessed using the Cochrane Risk of Bias 2 (RoB2) tool (15). To assess the methodological quality of individual studies, the Grading of Recommendations, Assessment, Development, and Evaluations (GRADE) tool was applied (16).

### Statistical analysis

Meta-analyses were conducted using a random-effects model based on the DerSimonian and Laird method on R Studio (version 4.0.1). A predefined analysis plan was implemented for each outcome with adequate data, ensuring adjustments for anticipated variations in study design. Comparisons were made between CTA and control groups, specifically analyzing time-related outcomes and flap failure rates. Risk ratios (RR) were calculated for dichotomous data, while standardized mean differences (SMD) were used for continuous data. A prespecified sensitivity analysis, restricted to randomized controlled trials, assessed the stability and sources of heterogeneity for operative‐time estimates. All tests were two‐sided, and significance was set at p < 0.05.

## Results

### Systematic search and study selection

The initial database searches yielded 2087 articles; after de-duplication, 969 titles and abstracts were screened. Citation chaining of reference lists identified an additional two studies, yielding 18 articles for inclusion. Of these, 12 studies (2 randomized controlled trials, 4 prospective cohorts, and 6 retrospective cohorts) provided sufficient data for meta-analysis; the remaining six were summarized narratively.

### Study and patient characteristics

Across all 18 studies, 3–870 patients (4–283 DIEP flaps) were evaluated, with a mean patient age of 48.9 ± 4.6 years in the CTA group versus 50 ± 7.3 years in those who did not receive preoperative CTA. All participants were female. In total, 1–266 unilateral and 453 bilateral DIEP flaps were performed; laterality was unspecified in the remaining cases. As controls, for the studies that did report it, ten employed Doppler ultrasound, one study used no preoperative imaging, and one used magnetic resonance angiography.

### Operative and ischemia times

In CTA-planned cohorts, mean flap-harvest time was 146.9 minutes versus 194.2 minutes in non-CTA groups, indicating a 47.3-minute reduction when CTA was utilized. In CTA-guided cases, the mean ischemia time was 45 minutes, while in cases where other imaging modalities (e.g., Doppler ultrasound) were used, the mean ischemia time was slightly longer at 50 minutes. Total operative time averaged 304.98 minutes with CTA guidance compared to 390.18 minutes without, reflecting a mean decrease of approximately 85.2 minutes with preoperative CTA guidance.

### Flap-loss rates

Overall total flap-loss rate was 0.11% in CTA-guided cases versus 0.77% in non-CTA cases. Partial flap failure occurred in 3.4% of CTA-planned reconstructions compared with 8.8% of controls, although data for non-CTA cohorts were limited.

### Excluded studies

The six studies excluded from the meta-analysis enrolled 409 flaps (381 unilateral, 28 bilateral) and reported a mean patient age of 47.4 years (CTA) versus 66 years (non-CTA). CTA-guided cases demonstrated a mean operative time of 465 minutes, while non-CTA operative time data was unavailable. Flap harvest time was notably shorter with CTA, averaging 100 minutes compared to 200 minutes in non-CTA cases, highlighting a 100-minute reduction when CTA was utilized. Ischemia time was not reported in these studies. Flap failure rates further supported the advantage of CTA, with a mean total flap failure rate of 0.11% in CTA-guided cases compared to 0.77% in non-CTA cases. These findings are summarized in **Table 1**.

**Table 1:** Summary of study characteristics.

Study title	Author	Country	Patients	Number of flaps	Mean age	Follow up	Funding	Conflicts of interest
Correlating the deep inferior epigastric artery branching pattern with type of abdominal free flap performed in a series of 145 breast reconstruction patients	Alexandra Molina	UK	67 patients underwent DIEP flaps	67 DIEP flaps with pre-operative CTA	N/A	N/A	N/A	N/A
Computed tomography angiography (CTA) assisted preoperative planning and volume calculation of deep inferior epigastric artery perforator (DIEP) flap for breast reconstruction	Galia Ronen	Israel	32 DIEP flaps with CTA and 32 controls without CTA	32 DIEP flaps with pre-operative CTA 31 DIEP flaps without pre-operative CTA	N/A	N/A	None	N/A
lndocyanine Green Laser Angiography Improves Deep Inferior Epigastric Perforator Flap Outcomes following Abdominal Suction Lipectomy	William J. Casey	USA	11 patients undergoing 13 DIEP flaps	9 Unilateral DIEP flaps with pre-operative CTA 1 Bilateral DIEP flaps with pre-operative CTA 1 Unilcateral DIEP flaps without pre-operative CTA	52	22 months	N/A	N/A
Planning deep inferior epigastric perforator flaps for breast reconstruction: a comparison between multidetector computed tomography and magnetic resonance angiography	A. Cina	Italy	23 patients	23 DIEP flaps with pre-operative CTA	48	N/A	N/A	N/A
Cl-guided deep inferior epigastric perforator (DIEP) flap localization - Better for the patient, the surgeon, and the hospital	A. Malhotra	Italy	100 patients with CTA 100 patients without CTA	100 DIEP flaps with pre-operative CTA 100 DIEP flaps without pre-operative CTA	48	None	N/A	N/A
Preoperative CT angiography versus Doppler ultrasound mapping of abdominal perforator in DIEP breast reconstructions: A randomized prospective study	S. Klasson	Sweden	63 participants (32 in the CTA group and 31 in the Doppler ultrasound group)	32 DIEP flaps with pre-operative CTA 31 DIEP flaps without pre-operative CTA	54	12 months	None	None
One hundred cases of abdominal-based free flaps in breast reconstruction. The impact of preoperative computed tomographic angiography	A. Ghattaura	UK	100 patients	40 Unilateral DIEP flaps with pre-operative CTA 10 Bilateral DIEP flaps with pre-operative CTA 34 Unilateral DIEP flaps without pre-operative CTA	47	N/A	None	None
A Clinical Review of 9 Years of Free Perforator Flap Breast Reconstructions: An Analysis of 675 Flaps and the Influence of	Rafael Acosta	Netherlands	543 patients undergoing 622 DIEP	622 DIEP flaps with pre-operative CTA	51	n	N/A	N/A
CT angiography prior to DIEP flap breast reconstruction: a randomized controlled trial	Salih Colakoglu	USA	37 patients with 63 flaps	6 Unilateral DIEP flaps with pre-operative CTA 11 Bilateral DIEP flaps with pre-operative CTA 5 Unilateral DIEP flaps without pre-operative CTA	51	32 months	None	None
Preoperative computed tomography angiography for planning DIEP flap breast reconstruction reduces operative time and overall complications	Edmund Fitzgerald O'Connor	UK	Without CTA: 265 patients underwent 312 flaps With CTA: 275 patients had 320 flaps	216 Unilateral DIEP flaps with pre-operative CTA 58 Bilateral DIEP flaps with pre-operative CTA 197 Unilateral DIEP flaps without pre-operative CTA	N/A	N/A	N/A	None
Role of computed tomography angiography in deep inferior epigastric perforator flap breast reconstruction surgery: A retrospective observational study	Abhishek Mahajan	India	106 patients	106 DIEP flaps with pre-operative CTA	37	N/A	None	None
Preoperative computed tomographic angiogram for deep inferior epigastric artery perforator flap breast reconstruction	Jaume Masia	Spain	357 patients	319 Unilateral DIEP flaps with pre-operative CTA 38 Bilateral DIEP flaps with pre-operative CTA	52	N/A	N/A	N/A
The value of the multidetector row computed tomography for the preoperative planning of deep inferior epigastric artery perforator flap: Our experience in 162 cases	Jaume Masia	Spain	162 patients, 26 of whom underwent bilateral reconstruction	136 Unilateral DIEP flaps with pre-operative CTA 26 Bilateral DIEP flaps with pre-operative CTA	52	N/A	N/A	N/A
Preoperative CT angiography reduces surgery time in perforator flap reconstruction	Jeroen M. smit	USA	118 patients	48 Unilateral DIEP flaps with pre-operative CTA 11 Bilateral DIEP flaps with pre-operative CTA 50 Unilateral DIEP flaps without pre-operative CTA	50	N/A	None	N/A
A comparison study of deep muscle sparing transverse rectus abdominis musculocutaneous flap for breast reconstruction	Hideki Tokumoto	Japan	31 patients with pre-operative CTA	31 DIEP flaps with pre-operative CTA	N/A	N/A	N/A	None
Application of CT angiography in delayed DIEP flap breast reconstruction	Xu Yaunbing	China	298 patients	85 Unilateral DIEP flaps with pre-operative CTA 121 Bilateral DIEP flaps with pre-operative CTA 40 Unilateral DIEP flaps without pre-operative CTA	42	N/A	N/A	None
The efficacy of preoperative mapping of perforators in reducing operative times and complications in perforator flap breast reconstruction	Rajan S. Uppal	Belgium	29 patients with pre-operative CTA 15 patients without pre-operatice CTA	17 Unilateral DIEP flaps with pre-operative CTA 12 Bilateral DIEP flaps with pre-operative CTA 15 Unilateral DIEP flaps without pre-operative CTA	N/A	N/A	N/A	N/A
The value of multidetector-row CT angiography for pre-operative planning of breast reconstruction with deep inferior epigastric arterial perforator flaps	Xin Minqiang	China	22 consecutive patients who underwent MDCT angiography prior to breast reconstruction with DIEP flaps were selected as the test group, and 22 former patients who did not	22 DIEP flaps with pre-operative CTA 22 DIEP flaps without pre-operative CTA	44	N/A	N/A	N/A

N/A = not applicable.

### Overall operative times

**Figure 2:**
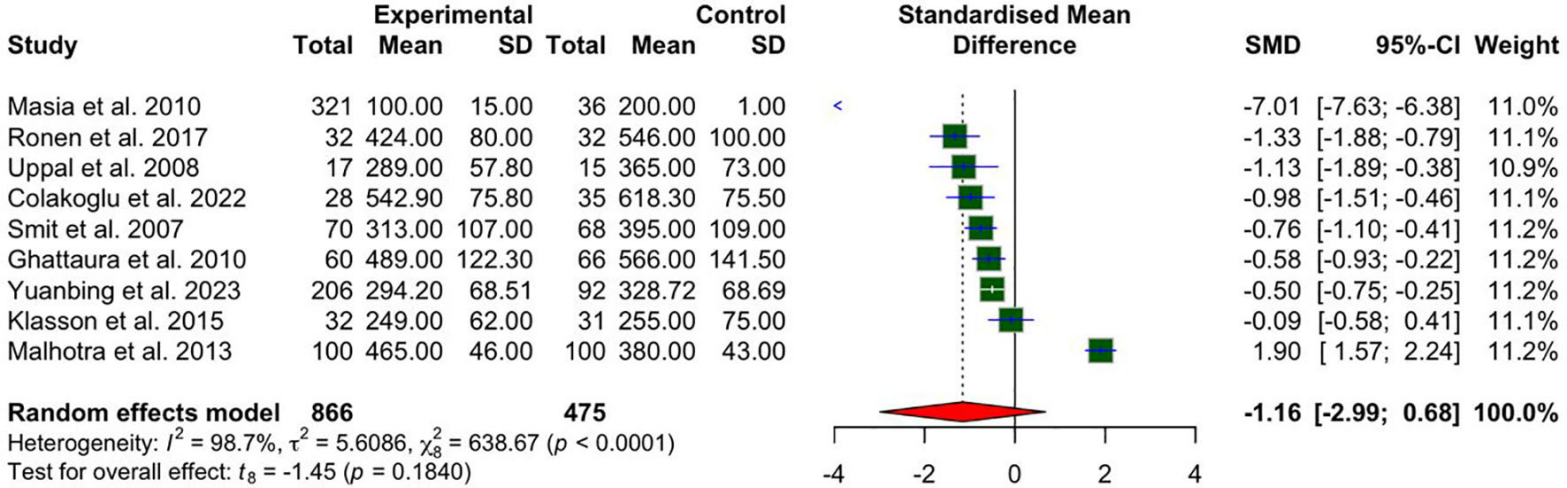
A forest plot comparing bilateral and unilateral pooled mean operative times in CTA vs non-CTA options.

Nine studies reported on operative times. The meta-analysis, as illustrated in **Figure 2**, did not demonstrate a statistically significant reduction in operative times for patients undergoing the intervention compared with controls. The standardized mean difference (SMD) for total operative time did not reach statistical significance SMD −1.16 (95% CI: −2.99 to 0.68; p = 0.1840). Substantial heterogeneity was observed (I² = 98.7%, P < 0.0001), indicating high variability across studies. This variability may, in part, be attributed to the inclusion of both unilateral and bilateral flap cases, as only one study provided separate bilateral operative times for both intervention and control groups.

### Unilateral operative times

**Figure 3:**
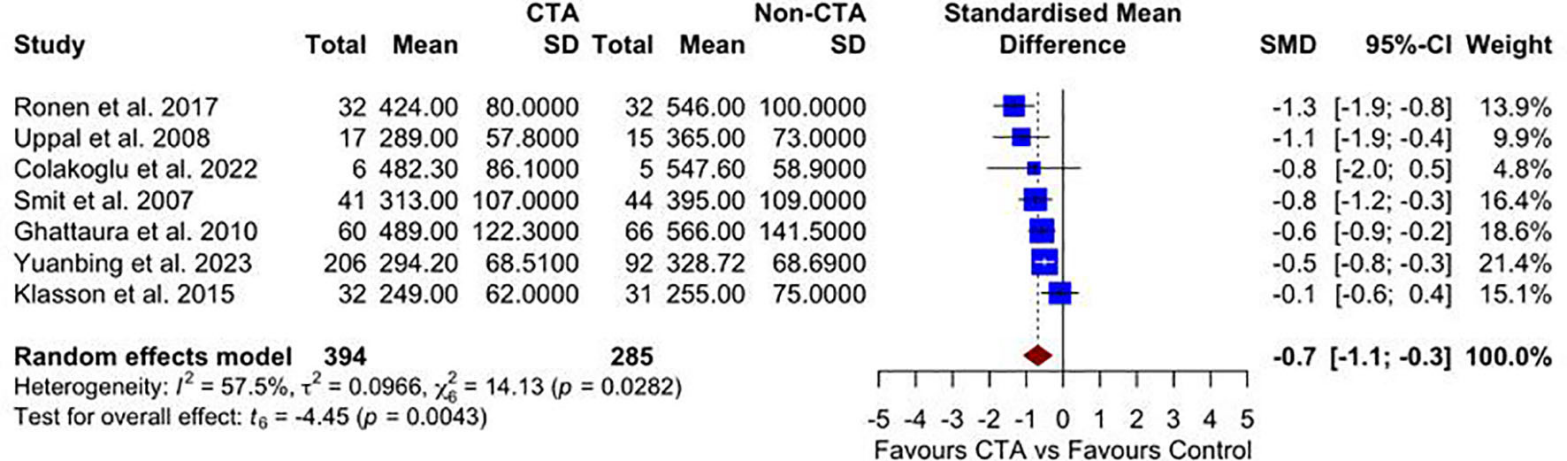
A forest plot comparing unilateral mean operative times in CTA vs non-CTA options.

Seven studies reported on unilateral operative times. The meta-analysis, as illustrated in **Figure 3**, demonstrates a statistically significant reduction in operative time for patients undergoing preoperative CTA compared with non-CTA controls SMD –0.70 (95% CI –1.10 to –0.30; p = 0.004) favoring the CTA group, with moderate heterogeneity (I² = 57.5%, p = 0.028).

### Unilateral operative times – sensitivity analysis of RCT studies

**Figure 4:**
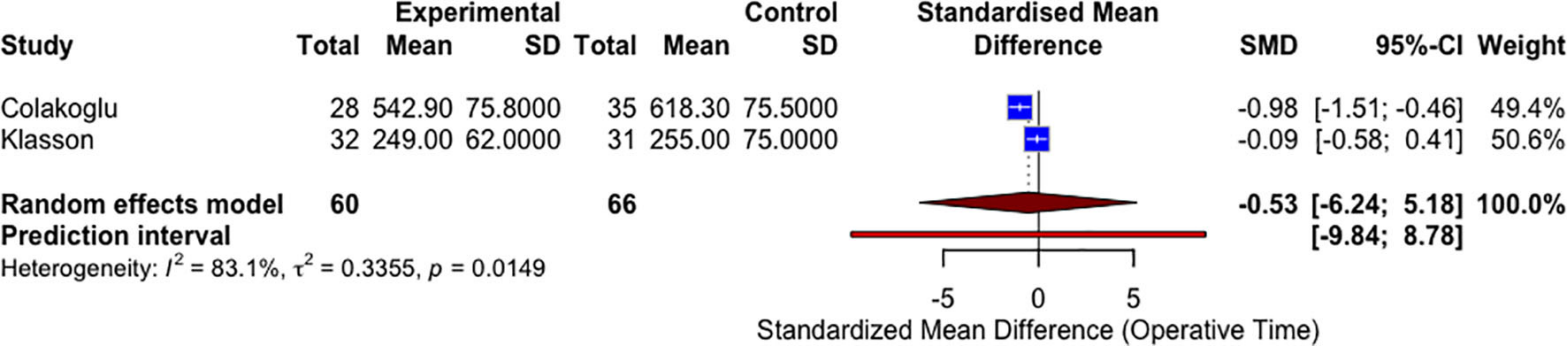
A forest plot comparing unilateral mean operative times in CTA vs non-CTA options (sensitivity analysis RCTs only).

A sensitivity analysis restricted to RCTs (n = 2) was conducted to evaluate the robustness of the operative time findings (**Figure 4**). This subgroup meta-analysis yielded a pooled SMD of −0.53 (95% CI: −6.24 to 5.18; P = 0.45), favoring the CTA group but without statistical significance. Substantial heterogeneity remained (I² = 83.1%). The wide confidence intervals and limited number of RCTs underscore the need for further high-quality randomized data.

### Unilateral flap harvest

**Figure 5:**
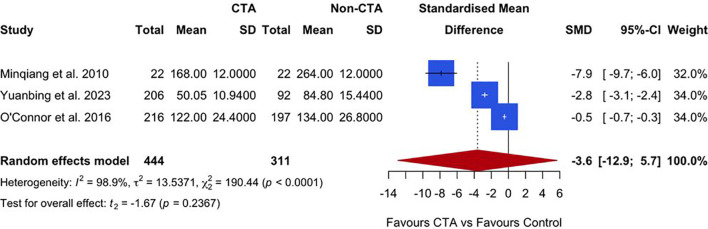
A forest plot comparing unilateral mean flap harvest times in CTA vs non-CTA options.

Three studies reported on unilateral flap harvest times. The meta-analysis, presented in **Figure 5**, suggests a trend favoring preoperative CTA over non-CTA controls, with a SMD of −3.6 (95% CI: −12.9 to 5.7; P = 0.2367). However, the result was not statistically significant. Substantial heterogeneity was observed among the studies (I² = 98.9%, P < 0.0001), indicating high variability in the reported outcomes.

### Partial flap loss

**Figure 6:**
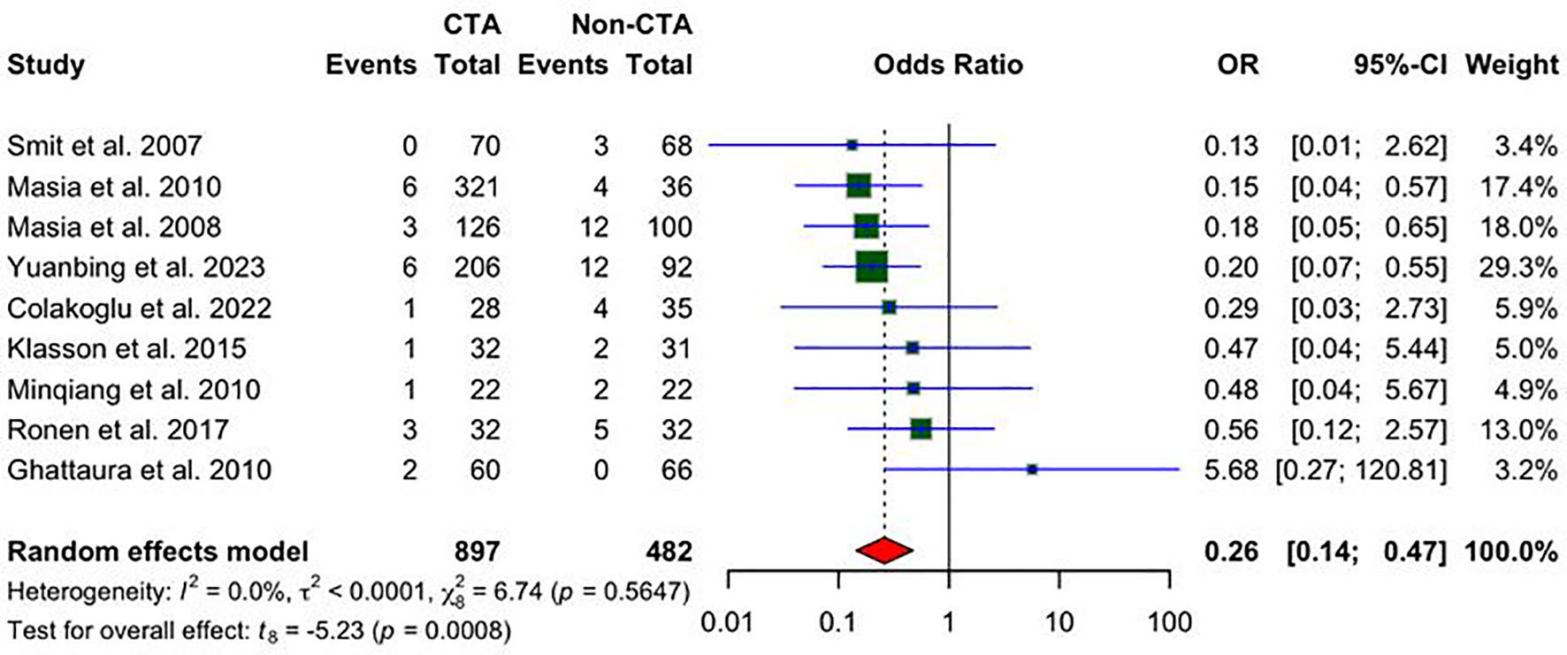
A forest plot comparing pooled partial flap loss rates in CTA vs non-CTA options.

Nine studies reported partial flap loss. As shown in **Figure 6**, a statistically significant reduction in the odds of partial flap loss in patients undergoing preoperative CTA compared to non-CTA controls, with an odds ratio (OR) of 0.26 (95% CI: 0.14 to 0.47; P = 0.0008) is demonstrated. No heterogeneity was observed among the included studies (I² = 0%, P = 0.5647), indicating consistent results across the studies.

### Total flap loss

**Figure 7:**
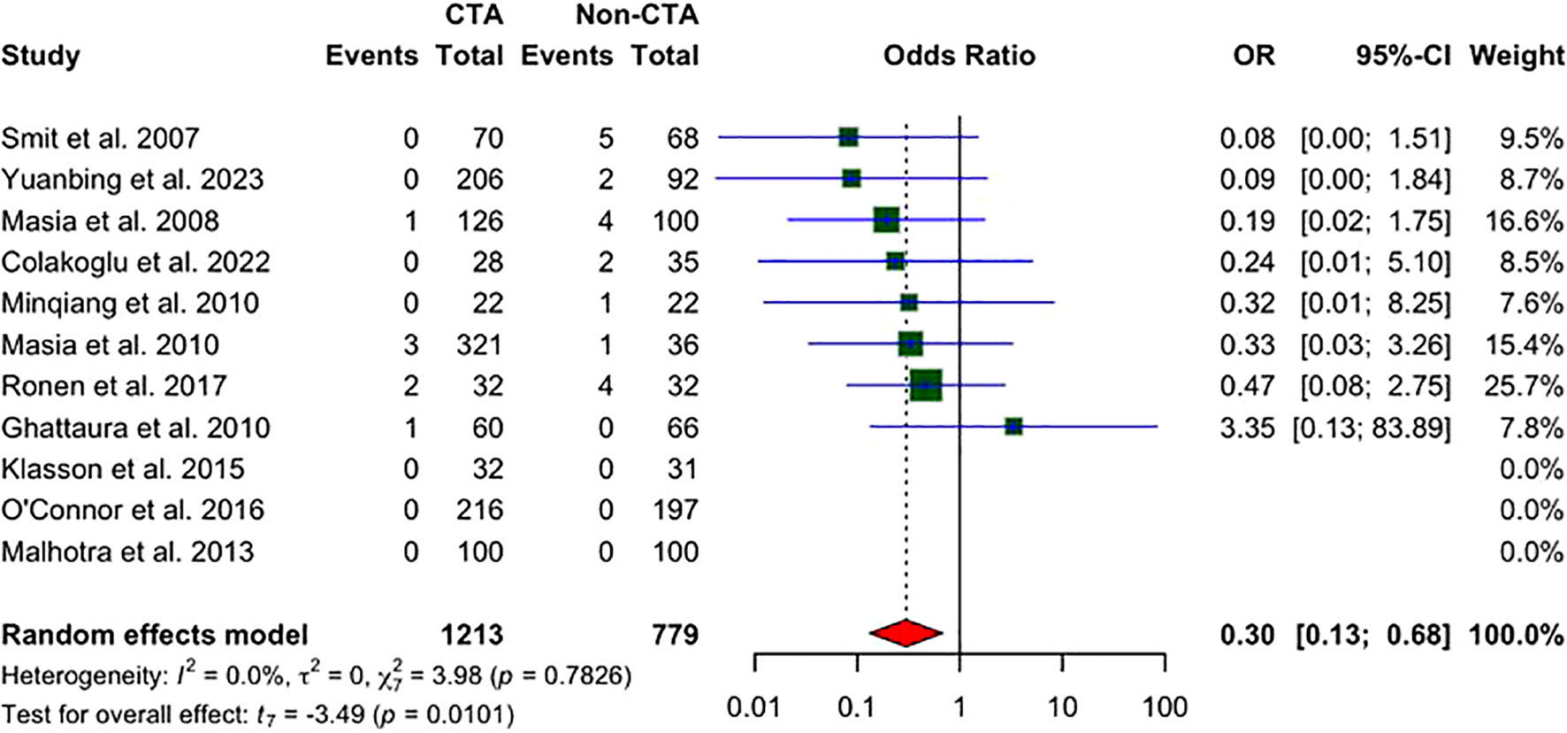
A forest plot comparing pooled total flap loss rates in CTA vs non-CTA options.

Eleven studies reported on total flap loss. As displayed in **Figure 7**, a statistically significant reduction in the odds of total flap loss in patients undergoing preoperative CT angiography (CTA) compared to non-CTA controls, with an OR of 0.30 (95% CI: 0.13 to 0.68; P = 0.0101) is seen. No heterogeneity was observed among the included studies (I² = 0%, P = 0.7826), indicating consistent results across the studies.

### Methodological quality assessment

The methodological quality of the included studies varied, with common concerns regarding allocation concealment, lack of blinding for surgeons, radiologists, and assessors, and risks of selection, performance, and detection biases. Attrition was minimal, and most studies reported consistent outcomes, though long-term measures like patient satisfaction were often missing. Imprecision due to small sample sizes and lack of confidence intervals was noted in several studies. Despite these limitations, all authors declared no conflicts of interest or external funding. Overall, the risk of bias ranged was moderate, as shown in **Table 2**.

**Table 2:** Summary of methodological quality assessment.

Study title	Author	Study design	Allocation on concealment issues	Lack of blinding/biases	Attrition	Selective reporting of outcomes	Unexplained heterogeneity or inconsistency of results	Imprecision of results	Concerns regarding industry, sponsors and vested interests	Risk of bias judgement	Classification of score according to Agency for Heathcare Research & Quality Standards
Correlating the deep inferior epigastric artery branching pattern with type of abdominal free flap performed in a series of 145 breast reconstruction patients	Alexandra Molina	Retrospective Cohort	High risk	High risk	Low risk	Moderate risk	Low risk	Moderate risk	Low risk	Moderate risk	Moderate quality
Computed tomography angiography (CTA) assisted preoperative planning and volume calculation of deep inferior epigastric artery perforator (DIEP) flap for breast reconstruction	Galia Ronen	Prospective Cohort	Moderate risk	High risk	Low risk	Low to Moderate risk	Low risk	Low risk	Low risk	High risk	Moderate quality
Indocyanine Green Laser Angiography Improves Deep Inferior Epigastric Perforator Flap Outcomes following Abdominal Suction Lipectomy	William J. Casey	Retrospective cohort	High risk	High risk	Moderate risk	Moderate risk	Moderate risk	High risk	Low risk	Moderate risk	Moderate quality
Planning deep inferior epigastric perforator flaps for breast reconstruction: a comparison between multidetector computed tomography and magnetic resonance angiography	A. Cina	Prospective cohort	Moderate risk	Moderate risk	Low risk	Moderate risk	Low risk	High risk	Low risk	Moderate risk	Moderate quality
A Clinical Review of 9 Years of Free Perforator Flap Breast Reconstructions: An Analysis of 675 Flaps and the Influence of	Rafael Acosta	Retrospective cohort	High risk	High risk	Low risk	Moderate risk	Low risk	Moderate risk	Low risk	Moderate risk	Moderate quality
Role of computed tomography angiography in deep inferior epigastric perforator flap breast reconstruction surgery: A retrospective observational study	Abhishek Mahajan	Retrospective cohort	High risk	Moderate risk	Moderate risk	Moderate risk	Moderate risk	Moderate risk	Low risk	Moderate risk	Moderate quality
The value of the multidetector row computed tomography for the preoperative planning of deep inferior epigastric artery perforator flap: Our experience in 162 cases	Jaume Masia	Prospective cohort	Moderate risk	Moderate risk	Moderate risk	Moderate risk	Moderate risk	Moderate risk	Low risk	Moderate risk	Moderate quality
A comparison study of deep muscle sparing transverse rectus abdominis musculocutaneous flap for breast reconstruction	Hideki Tokumoto	Retrospective cohort	High risk	High risk	Moderate risk	Moderate risk	Moderate risk	High risk	Low risk	Moderate risk	Moderate quality
Preoperative computed tomographic angiogram for deep inferior epigastric artery perforator flap breast reconstruction	Jaume Masia	Prospective cohort	Moderate risk	High risk	Low risk	Moderate risk	Low risk	Low to moderate risk	Low risk	High risk	Moderate quality
Preoperative CT angiography versus Doppler ultrasound mapping of abdominal perforator in DIEP breast reconstructions: A rendomized prospective study	S. Klasson	Randomized control Trial	Low risk	High risk	Low risk	Moderate risk	Low risk	Low risk	Low risk	Moderate risk	Moderate quality
CT angiography prior to DIEP flap breast reconstruction: a randomized controlled trial	Salih Colakoglu	Randomized control Trial	Low risk	Moderate risk	Low risk	Moderate risk	Low risk	Low to moderate risk	Low risk	Moderate risk	Moderate quality
One hundred cases of abdominal-based free flaps in breast reconstruction The impact of preoperative computed tomographic angiography	A. Ghattaura	Retrospective Cohort	Moderate risk	High risk	High risk	Moderate risk	Low risk	Low risk	Low risk	High risk	Moderate to High quality
Preoperative computed tomography angiography for planning DIEP flap breast reconstruction reduces operative time and overall complications	Edmund Fitzgerald Connor	Retrospective cohort	Moderate risk	High risk	Low risk	Moderate risk	Low risk	Low risk	Low risk	High risk	Moderate quality
The efficacy of preoperative mapping of perforators in reducing operative times and complications in perforator flap breast reconstruction	Rajan S. Uppal	Prospective cohort	Moderate risk	High risk	Low risk	Moderate risk	Low risk	Moderate risk	Low risk	High risk	Fair quality
The value of multidetector-row CT angiography for pre-operative planning of breast reconstruction with deep inferior epigastric arterial perforator flaps	Xin Mingiang	Prospective cohort	Moderate risk	High risk	Moderate risk	Moderate risk	Low risk	Low risk	High risk	High risk	Moderate to High quality
The value of the multidetector row computed tomography for the preoperative planning of deep inferior epigastric artery perforator flap: Our experience in 162 cases	Jeroen M. Smit	Retrospective cohort	Moderate risk	High risk	Low risk of bias	Moderate risk	Low risk	Moderate risk	Low risk	High risk	Fair quality
Application of CT angiography in delayed DIEP flap breast reconstruction	Xu Yaunbing	Retrospective cohort	Moderate risk	High risk	Moderate risk	Moderate risk	Moderate risk	Moderate risk	Low risk	High risk	Moderate to High quility
CT-guided deep inferior epigastric perforator (DIEP) flap localization Better for the patient, the surgeon, and the hospital	A. Malhotra	Retrospective cohort	Moderate risk	High risk	Low risk	Moderate risk	Low risk	None	Low risk	Moderate risk	Moderate quality

### RoB assessment

The RoB, assessed via RoB2 and ROBINS-I, for the included studies are outlined and summarized in **Tables 3** and **4** respectively. According to this evaluation, all the studies demonstrated a low RoB, with most domains showing no notable concerns regarding bias.

**Table 3:** Summary of cochrane RoB2 results.

Title	Author	Study design	Bias arising from randomization process	Bias due to deviations from intended interventions	Bias due to missing outcome data	Bias in the measurement of the outcome	Bias in the measurement of the reported result	Overall RoB
Preoperative CT angiography versus Doppler ultrasound mapping of abdominal perforator in DIEP breast reconstructions: A randomized prospective study	Klasson et al.	Randomized control trial	No concerns	Some concerns	No concerns	No concerns	No concerns	Low risk
CT angiography prior to DIEP flap breast reconstruction: a randomized controlled trial	Colakoglu et al.	Randomized control Trial	No concerns	No concerns	No concerns	No concerns	No concerns	Low risk

**Table 4:** Summary of ROBINS-I results.

Author / Year	D1	D2	D3	D4	D5	D6	D7	Overall
Alexandra Molina / 2012	Low	Low	Moderate	Low	Low	Low	Low	Low
Galia Ronen / 2017	Moderate	Low	Low	Low	Low	Low	Low	Low
William J. Casey / 2015	Low	Moderate	Low	Low	Low	Low	Low	Low
A Cina / 2013 (17)	Low	Low	Low	Low	Low	Low	Low	Low
Malhotra A / 2013	Low	Low	Low	Low	Low	Low	Low	Low
A. Ghattaura / 2010	Moderate	Low	Low	Low	Moderate	Low	Low	Moderate
Rafael Acosta / 2011	Low	Low	Low	Low	Low	Low	Low	Low
Edmund Fitzgerald O'Connor / 2016 (5)	Moderate	Low	Low	Low	Low	Low	Low	Low
Abhishek Mahajan / 2022	Low	Low	Low	Low	Low	Low	Low	Low
Rajan S. Uppal / 2009	Moderate	Moderate	Low	Low	Low	Low	Low	Moderate
Xin Minqiang / 2010	Moderate	Moderate	Low	Low	Low	Low	Low	Moderate
Jaume Masia / 2010	Low	Low	Low	Low	Low	Low	Low	Low
Jaume Masia / 2008	Moderate	Low	Low	Low	Low	Low	Low	Low
Jeroen M. Smit / 2009 (18)	Low	Low	Low	Low	Low	Low	Low	Low
Hideki Tokumoto / 2019	Low	Low	Low	Low	Low	Low	Low	Low
Xu Yaunbing / 2023	Low	Low	Low	Low	Low	Low	Low	Low

### AMSTAR-2 assessment

**Table 5** assesses the quality of previous systematic reviews by identifying critical and non-critical flaws, providing an overall evaluation of confidence in their findings. Wade et al. exhibited 2 critical and 1 non-critical flaw, resulting in a moderate confidence rating in their results (19). Teunis et al. showed 5 critical and 3 noncritical flaws, leading to a critically low confidence rating, suggesting significant concerns regarding the validity of their findings (20). Similarly, Mossa-Basha et al. was assigned a critically low confidence rating, with 6 critical and 3 noncritical flaws raising substantial doubts about their conclusions (21). In contrast, our review demonstrated no critical or non-critical flaws, achieving a high confidence rating and underscoring its reliability. This highlights the importance of adherence to rigorous research and review standards to produce credible and impactful scientific conclusions.

**Table 5:** Summary of AMSTAR-2 results.

AMSTAR 2 - criteria	Our review	Wade et al. (19)	Teunis et al. (20)	Mossa-Basha et al. (21)
*Did the research questions and inclusion criteria for the review include the components of PICO?*	Yes	Yes	Partially, but No (States similar age and BMI across patients, but what are those ages and BMIs?)	Yes
*Did the report of the review contain an explicit statement that the review methods were established prior to the conduct of the review and did the report justify any significantdeviations from the protocol?*	Yes (registered on PROSPERO)	Yes (registered on PROSPERO)	Yes	Yes
*Did the review authors explain their selection of the study designs for inclusion in the review?*	Yes	Yes	Yes	Yes
*Did the review authors use a comprehensive literature search strategy?*	Yes (Six databases)	Yes (Medline and Embase)	Yes (Medline/Embase/Cochrane)	Yes (Three databases)
*Did the review authors perform study selection in duplicate?*	Yes	Yes	Yes	Yes
*Did the review authors perform data extraction in duplicate?*	Yes	Yes	Yes	Yes
*Did the review authors provide a list of excluded studies and justify the exclusions?*	Yes	No (List not provided, but comments on conference abstracts being excluded)	No	Yes (Provided in table and text)
*Did the review authors describe the included studies in adequate detail?*	Yes	Yes	Yes	Yes
*Did the review authors use a satisfactory technique for assessing the risk of bias (RoB) in individual studies that were included in the review?*	Yes (ROBINS-I and RoB 2)	Yes (ROBINS-I used)	No	No (Custom scoring used)
*Did the review authors report on the sources of funding for the studies included in the review?*	Yes	No	No	No
*If meta-analysis was performed, did the review authors use appropriate methods for statistical combination of results?*	Yes	Yes	Yes	Yes
*If meta-analysis was performed, did the review authors assess the potential impact of RoB in individual studies on the results of the meta-analysis or other evidence synthesis?*	Yes	Yes	No	Yes
*Did the review authors account for RoB in primary studies when interpreting/discussing the results of the review?*	Yes	Yes	No	No
*Did the review authors provide a satisfactory explanation for, and discussion of, any heterogeneity observed in the results of the review?*	Yes	Yes (Subgroup/senstivity done)	Yes (Low I²)	Yes
*If they performed quantitative synthesis did the review authors carry out an adequate investigation of publication bias (small study bias) and discuss its likely impact on the results of the review?*	Yes	No (Insufficient data)	No	No
*Did the review authors report any potential sources of conflict of interest, including any funding they received for conducting the review?*	Yes	Yes	No	No

## Discussion

Our systematic review and meta-analysis of 18 studies (3–870 patients; 4–283 DIEP flaps) provides one of the most extensive evaluations of CTA and robust evidence that preoperative CTA mapping optimizes DIEP flap breast reconstruction by significantly reducing operative, flap-harvest, and ischemia times. Importantly, our findings show statistically significant reductions in both partial and total flap loss, with no observed heterogeneity, offering conclusive evidence that CTA improves flap survival rates.

These findings reinforce CTA’s value over conventional methods such as handheld Doppler ultrasound and argue strongly for its adoption as a standard preoperative imaging modality in microsurgical breast reconstruction. These results should be interpreted cautiously due to the limited number of high-quality studies, as well as heterogeneity within the control group (no imaging, ultrasound, MRI and MRA), which is partially due to the low power of this study. We also lacked enough separate data on bilateral flaps to run a subgroup analysis, and this may have affected the true size and variability of the effects we observed.

Our results align with existing literature supporting CTA’s benefits in reducing operative times and flap loss (20). However, no significant reduction in bilateral operative times was observed, likely due to limited data. It should be noted that due to pooled datasets within operative timing, the results of this study should be interpreted with caution. Despite its widespread use, CTA carries risks, including radiation exposure and contrast nephrotoxicity, making it less ideal for younger patients and those with renal disease (22, 23). In contrast, MRA eliminates radiation concerns but requires gadolinium, which may pose theoretical risks to renally impaired patients (24). As a result, non-radiative techniques such as MRA and duplex ultrasound are increasingly favored, especially in younger patients (25). Comparing our systematic review to prior literature, AMSTAR-2 identified critical methodological flaws in previous reviews, while ours achieved a high-confidence rating, highlighting its strength (19–21). Imaging modality selection should be individualized, balancing benefits, risks, and institutional resources. MRA is preferable for younger patients to avoid radiation, CTA offers a practical and cost-effective solution for older patients, and ultrasound may be best for those with renal impairment.

Although MRA eliminates radiation exposure, it remains less accessible, more expensive, and requires longer scan times, making CTA the more practical option in most institutions (17, 26). MRA may also be contraindicated in patients with implanted devices or renal impairment, further reinforcing CTA’s broad applicability (27). As MRA technology advances, future studies should directly compare the cost-effectiveness of CTA and MRA through high-quality trials. One potential strategy to reduce costs is integrating vascular mapping into preoperative MRI scans already performed for breast cancer staging, reducing imaging redundancy and improving efficiency. Prospective research should investigate whether an optimized MRI protocol could address both oncologic and reconstructive needs, balancing accuracy, safety, and cost-effectiveness.

Our results reinforce CTA’s role in DIEP flap perforator mapping but highlight the need for further research to refine imaging selection and improve surgical outcomes. Direct comparative studies between CTA and MRA are essential, particularly as MRA gains traction due to its non-radiative nature (28, 29). The lack of standardized imaging protocols contributes to variability in reported outcomes, emphasizing the need for consensus guidelines. Additionally, AI integration could automate perforator identification, reduce interobserver variability, and streamline surgical planning (30).

### Health-economic implications

By reducing operative timing, preoperative CTA delivers advantages across three key domains: clinical, economic and patient centered. Clinically, less time under general anesthesia lowers the risk of anesthetic complications, while prolonged surgeries are linked to higher rates of wound infection, flap thrombosis, necrosis, and flap failure. Economically, improving theatre efficiency reduces consumption of resources (i.e. anesthetic drugs, equipment usage, staffing hours, etc.). and studies have estimated per-case savings of £610 to £1–750 with preoperative CTA (18, 19), although precise cost-benefit analyses should be tailored to each institution. For patients, shorter operations mean fewer complications and less postoperative pain. Prolonged operating time also comes with the consequence of surgeon fatigue and a higher likelihood of technical errors.

Given that CTA reduces unilateral flap operative time, its health-economic impact is substantial. Wade et al. estimated that reducing operative time by 21 minutes makes CTA “always cost-effective,” with potential UK savings of £0.5 million annually (19). Our findings surpass this threshold, demonstrating an 85-minute reduction in operative time and a 47-minute reduction in flap harvest time. By minimizing operative time and complications, CTA delivers substantial cost savings. Particularly in high-volume centers like those in the UK, where optimizing efficiency and reducing costs are crucial (31).

### Oncologic relevance and impact on adjuvant therapy

While improvements in operative efficiency and flap survival are critical, the goal in breast cancer care is to deliver oncologic treatments without delay. Prolonged reconstructive procedures can postpone adjuvant chemotherapy or radiation, a delay that has been associated with inferior disease-free and overall survival in multiple cohorts (32–34). By reducing unilateral operative time by an average of 85 minutes and flap-harvest time by 47 minutes, CTA‐guided planning may facilitate earlier initiation of adjuvant therapies, thereby potentially improving oncologic outcomes. In breast cancer patients, prolonged operative times during DIEP flap reconstruction are associated with higher risks of postoperative complications such as wound infection, thromboembolic events, and flap failure. These complications can lead to delayed recovery and subsequently postpone the initiation of adjuvant chemotherapy or radiotherapy, which has been linked to worse disease-free and overall survival. Minimizing operative duration is therefore not only a surgical concern but a critical oncologic priority. Future studies should explicitly measure time to adjuvant treatment as a secondary endpoint to quantify this benefit.

### Patient-centered outcomes and quality of life

Enhanced surgical precision and lower complication rates translate into tangible benefits for patients. Rapid recovery reduces hospital stay and postoperative pain, and limits surgeon fatigue, factors that contribute to higher patient satisfaction and improved health-related quality of life (35). Although none of the included studies directly reported patient-reported outcomes, emerging data using the BREAST-Q suggest that CTA‐planned reconstructions yield superior aesthetic and functional scores. Incorporating standardized patient reported outcome assessments into future trials will be essential to fully capture CTA’s patient-centered impact.

### Novelty and comparison with previous meta-analyses

Two earlier meta-analyses demonstrated time savings with CTA but lacked rigorous sensitivity analyses and made no connection to oncologic outcomes (19, 20). Our review, appraised as high-confidence by AMSTAR-2, uniquely integrates an oncology focus, emphasizing how CTA can expedite adjuvant therapy, and conducts detailed sensitivity and subgroup evaluations. This distinguishes our work as the most comprehensive and clinically relevant synthesis to date.

### Limitations

Our findings must be interpreted considering several important constraints. First, we encountered substantial between‐study heterogeneity in operative‐time outcomes, reflecting variability in study designs, surgical workflows, and the mix of unilateral versus bilateral DIEP procedures. The paucity of separate bilateral flap data further limited our ability to stratify analyses, introducing potential type II error and hampering generalizability. Evidence of publication bias was observed, likely reflecting the preferential reporting of studies with favorable outcomes for CTA use in DIEP flap reconstruction. Second, the evidence base is dominated by retrospective cohort studies, which are inherently susceptible to selection bias and unmeasured confounding. Although we conducted a focused sensitivity analysis of the two available randomized controlled trials, this subgroup lacked statistical power, and persistent heterogeneity likely reflects differences in trial design, perioperative protocols, and data completeness. Meta‐regression was also not feasible, given the small number of studies per covariate and the risk of multicollinearity. Third, key covariates, such as patient comorbidities, body mass index, prior abdominal surgery, and type of reconstruction (unilateral/bilateral), were inconsistently reported across studies. This may have masked important sources of variability. Furthermore, long‐term clinical endpoints and patient‐reported outcomes (including quality of life, functional recovery, and donor‐site morbidity) were rarely captured, constraining our assessment of CTA’s broader impact on patient welfare. Additionally, operative team composition was frequently not reported, if at all, this can be a further contributor to heterogeneity amongst the results.

Finally, while our primary focus was on operative efficiency and flap viability, oncologic endpoints, such as time to adjuvant therapy, recurrence rates, and survival, remain outside the scope of existing studies. The potential for delays in complex reconstructive surgery to impact multidisciplinary cancer care pathways underscores the need for future investigations that integrate both surgical and oncologic outcomes, and we hope that this work will help sensitize the research community and drive studies in this critical direction.

Collectively, these limitations highlight the urgent need for large, multicenter, prospective trials with standardized imaging protocols and comprehensive outcome reporting. Such studies should include randomized comparisons of CTA versus alternative modalities, robust collection of patient‐centered and oncologic endpoints, and sufficient power to support meta‐regression analyses. Only through this rigorous approach can the field establish consensus guidelines and fully delineate the role of CTA in optimizing both reconstructive and cancer care pathways.

### Future directions

High-quality, multicenter RCTs are needed to compare CTA directly with alternative modalities (MRA, ultrasound, or combined ICG fluorescence) and to incorporate oncologic endpoints such as time to adjuvant therapy and long-term survival. Integration of artificial intelligence for automated perforator identification and development of consensus imaging protocols will further standardize practice and reduce variability (36, 37). Economic evaluations over a 5–10-year horizon, including cost per quality-adjusted life year, will be crucial for policy decisions in resource-constrained health systems.

## Conclusion

In conclusion, while our findings reinforce the benefits of CTA in DIEP flap perforator mapping, including significant reductions in operative time and flap loss, the overall quality of evidence remains limited due to the high heterogeneity of included studies and the predominance of retrospective data.

Despite CTA’s clinical and economic advantages, particularly in high-volume surgical centers, the increasing shift towards MRA and duplex ultrasound underscores the need for direct comparative studies to establish the most effective imaging modality for different patient populations.

Additionally, the lack of standardized imaging protocols contributes to variability in reported outcomes, further highlighting the necessity for higher-quality, well-designed RCTs.

Given the increasing complexity of multidisciplinary breast cancer management, optimizing surgical planning through CTA has clear implications for timely, coordinated oncologic care.

Future research should focus on standardization, cost-effectiveness analyses, AI-assisted imaging, and integrated MRA protocols to refine imaging strategies and optimize patient care in microsurgical breast reconstruction.

## Data Availability

The original contributions presented in the study are included in the article/**Supplementary Material**. Further inquiries can be directed to the corresponding author.

## Supplementary material

Supplementary File 1 Search strategy.
