# Supplementary material for: A systematic review and meta-analysis on computed tomography angiography mapping for deep inferior epigastric perforator flap breast reconstruction
Source: Front Oncol. 2025 Sep 17;15:1600476. doi: 10.3389/fonc.2025.1600476 (PMC12483871; doi:10.3389/fonc.2025.1600476)
Supplement: Supplementary File 1 — Search strategy. [file DataSheet1.docx]

STEM 1 (DIEP): "perforator*" OR “perforator flap*” OR "perforating arter*" OR "perforating vessel*" OR “deep inferior epigastric perforator*” OR “deep inferior epigastric perforator flap*” OR “DIEP” OR “DIEP flap*” OR “flap*” OR “surgical flap*” OR “epigastric arter*” OR “abdominal perforator flap*” OR “epigastric arter* for surgical planning”

STEM 2 (Breast reconstruction): “breast reconstruction*” OR “mammoplast*” OR “mammaplast*”

STEM 3 (CTA): "mapping*" OR "preoperative mapping*" OR “perforator mapping*” OR "computed tomography angiograph*" OR "computed tomograph*" OR "angiograph*" OR “CT” OR “CTA”

STEM 4 (Outcomes: operative time, flap failure rates): “surgical outcome*” OR “operative outcome*” OR “clinical outcomes*” OR “operative tim*” OR “surgical tim*” OR “operative duration*” OR "procedure tim*” OR "tim*" OR “complication*” OR “failure*” OR “length of surger*” OR “CROM*”
